# Further Improvements of the *P. falciparum* Humanized Mouse Model

**DOI:** 10.1371/journal.pone.0018045

**Published:** 2011-03-31

**Authors:** Ludovic Arnold, Rajeev Kumar Tyagi, Pedro Meija, Claire Swetman, James Gleeson, Jean-Louis Pérignon, Pierre Druilhe

**Affiliations:** Malaria Vaccine Development Laboratory, Institut Pasteur, Paris, France; Agency for Science, Technology and Research - Singapore Immunology Network, Singapore

## Abstract

**Background:**

It has been shown previously that it is possible to obtain growth of *Plasmodium falciparum* in human erythrocytes grafted in mice lacking adaptive immune responses by controlling, to a certain extent, innate defences with liposomes containing clodronate (clo-lip). However, the reproducibility of those models is limited, with only a proportion of animals supporting longstanding parasitemia, due to strong inflammation induced by *P. falciparum.* Optimisation of the model is much needed for the study of new anti-malarial drugs, drug combinations, and candidate vaccines.

**Materials/Methods:**

We investigated the possibility of improving previous models by employing the intravenous route (IV) for delivery of both human erythrocytes (huRBC) and *P. falciparum,* instead of the intraperitoneal route (IP), by testing various immunosuppressive drugs that might help to control innate mouse defences, and by exploring the potential benefits of using immunodeficient mice with additional genetic defects, such as those with IL-2Rγ deficiency (NSG mice).

**Results:**

We demonstrate here the role of aging, of inosine and of the IL-2 receptor γ mutation in controlling *P. falciparum* induced inflammation. IV delivery of huRBC and *P. falciparum* in clo-lip treated NSG mice led to successful infection in 100% of inoculated mice, rapid rise of parasitemia to high levels (up to 40%), long-lasting parasitemia, and consistent results from mouse-to-mouse. Characteristics were closer to human infection than in previous models, with evidence of synchronisation, partial sequestration, and receptivity to various *P. falciparum* strains without preliminary adaptation. However, results show that a major IL-12p70 inflammatory response remains prevalent.

**Conclusion:**

The combination of the NSG mouse, clodronate loaded liposomes, and IV delivery of huRBC has produced a reliable and more relevant model that better meets the needs of Malaria research.

## Introduction

The development of a small laboratory model capable of tolerating and sustaining human malaria infection has almost unlimited applications in areas such as parasite biology, novel drug development and vaccine discovery.

Currently, the majority of *in vivo* investigations into malaria biology are performed using rodent malaria species such as *P. berghei,* and *P. yoelii* that are much easier to handle however, their relevance to human malaria has been questioned [1], [2], [3]. A convenient model capable of sustaining *P. falciparum* would undoubtedly be beneficial. For example, such a model could serve to harmonise *in vitro* studies that use *P. falciparum* in culture, with the *in vivo* models that presently use rodent species. While these approaches are complementary, the current mismatch of *Plasmodium* species is a very serious limitation. With the development of resistance against all existing anti-malarial drugs, the need for better tools to discover and develop novel classes and combinations of anti-malarials is imperative [4], [5].

The advent of several new mouse strains with genetic immune deficiencies has greatly benefited the development of a small laboratory malaria model, and results have shown that such a model is indeed both achievable and useful [6], [7], [8], [9], [10]. Experiments performed so far have used mouse strains such as SCID, NIH III (Beige Xid Nude), and NOD/SCID, together with pharmacological agents to control their remaining innate defences, or mouse adapted parasites. It was shown that by grafting them with either human erythrocytes [6], [11] or human hepatocytes [12] these animals can support, respectively, the asexual blood cycle, or the intra-hepatic cycle of the human parasite *P. falciparum*. However, on a day-to-day basis they are quite cumbersome to manage [6], [11], and this onus, combined with their currently poor reproducibility of successful infection, limits their usefulness, and is likely to have contributed to the persistent use of rodent *Plasmodium* species in the majority of experimental malaria studies - for reasons of convenience rather than scientific merit.

A main barrier to achieving an improved, workable *P. falciparum* mouse model is the strong pro-inflammatory effect of the parasite itself. In humans the asexual erythrocytic stages of *P. falciparum* are known to result in a systemic inflammatory process that is responsible for many of the symptoms of the disease [13]. Although the two situations differ in several respects, a similar inflammatory response was observed in immunodeficient mice, lacking T and B cells. In this case, as recently described [6], *P. falciparum* triggers strong pro-inflammatory reactions in monocytes and macrophages, contributing to partial control of the parasite and the human erythrocyte (huRBC) graft.

A second significant practical problem with all existing models developed to date is that huRBC are injected by the intra-peritoneal (IP) route, which relies on the successful migration of huRBC into the blood stream across the peritoneum. This is a process that is not properly understood, and, in turn, this prevents any rational analysis and improvement of the model. Existing models have been useful in proving the concept that *P.falciparum* can survive in a small rodent model, however they have also shown that unless inordinate volumes of blood are injected IP daily [11], [14], trans-peritoneal passage to the peripheral blood is uneven, particularly in the long term. This leads to important variations from one animal to the other, most likely related to the inflammatory reaction triggered by high parasite loads, as shown previously [6].

With these limitations of current models in mind, we thought it was essential to attempt to improve the *P. falciparum* humanised mouse model, particularly in terms of control of inflammatory reactions, and reproducibility of parasitemia. We decided to address these issues by using the IV route for huRBC and parasite administration, and by investigating other means to increase control over the mouse innate immune response. The use of this IV model led us to identify, among several factors investigated, the effect of aging and that of inosine as significant in reducing inflammatory reactions, and therefore improving *P. falciparum* growth. Moreover, after using various strains of immunodeficient mice, we investigated, as others [14], the value of the NOD/SCID/IL-2Rγ-null mouse (NSG mouse) which, due to the knock out of the γ-chain of the IL-2 receptor, has been shown to better tolerate a variety of transplanted human cells [15], [16], [17], [18].

The resulting new IV model using NSG mice presents several significant advantages over previously available models. It offers greater reproducibility, with 100% of mice successfully grafted without the need for mouse-adapted parasites, consistent curves of parasitemia, and high levels of infection with up to 40–50% of total erythrocytes infected.

## Materials and Methods

### 1. Mice

All procedures were carried out in line with the European Community Council Directive, 24^th^ November 1986 (86/609/EEC), and the European Union guidelines. All procedures were reviewed and approved by the Pasteur Institut Animal ethical committee (approval number A 75 15–27). Every effort were made to minimize suffering.

BALB/c, NOD/SCID and NSG mice were purchased from Charles River. Immunodeficient mice were kept in sterile isolators. They were housed in sterilized cages equipped with filter tops during the experimentation, and they were provided with autoclaved tap water and a γ-irradiated pelleted diet *ad libitum*. They were manipulated under pathogen free conditions using laminar flux hoods.

### 2. Human Red Blood Cells (huRBC)

Human whole blood was provided by the French blood bank (Etablissement Français du Sang, Paris, France) and used in accordance with French legislation. Blood donors had no history of malaria and all blood-groups were used without observing any difference on parasite survival. Whole blood was centrifuged thrice at 900×g, for 5 minutes at room temperature and the buffy coat was separated in order to eliminate white blood cells and platelets. Packed RBC were suspended in SAGM (Adenine, Glucose and mannitol solution) and kept at 4°C for a maximum of 2 weeks. Before use, huRBC were washed three times with RPMI-1640 medium (Gibco/BRL, Grand Island, N.Y.), supplemented with 1 mg of hypoxanthine per liter (Sigma, St Louis, MO) and warmed at 37°C.

Blood samples drawn from mice were used to determine the percentage of huRBC in mouse peripheral blood at regular intervals by flow cytometry on a FACScalibur (BD biosciences) using FITC labeled anti-human glycophorin monoclonal antibody (Dako, Denmark).

### 3. Parasites

*P. falciparum* lines 3D7, UPA, and K1 were employed in this study, along with one clinical isolate taken from a patient at Bichat Hospital, Paris (used the day after sampling). The Uganda Palo Alto (UPA) strain employed was the Palo Alto Marburg line, this was used for all experiments conducted unless otherwise specified. Parasite cultures were not synchronized and therefore a mix of various developmental stages was injected to infect mice. Parasites were maintained under *in vitro* conditions at 5% hematocrit at 37°C in a candle jar in complete culture medium (RPMI-1640 medium (Gibco/BRL), 35 mM HEPES (Sigma), 24 mM NaHCO_3_, 10% albumax (Gibco/BRL) and 1 mg of hypoxanthine (Sigma) per liter. Parasite samples were cryopreserved using the glycerol/sorbitol method [19]. The cultures were controlled for *Mycoplasma* contamination by using PCR testing.

### 4. Mouse infection and immunomodulation protocol

NOD/SCID mice were retro-orbitally injected with 400 µl huRBC every 3 days to ensure a satisfactory proportion of huRBC (i.e. chimerism), at the time of infection (≈60%). Simultaneously, 0.1 ml of unsized dichloromethylene diphosphonate (Cl_2_MDP) encapsulated in liposome (clo-lip) (provided by Nico Van Rooijen) diluted in 0.4 ml RPMI was intraperitoneally injected. Four injections at 2–3 day intervals were given before parasite infection. At the time of the fifth injection, mice were retro-orbitally injected with 300 µl of a *P. falciparum* infected huRBC suspension in RPMI at a parasitemia of 1% (all the developmental forms, *i.e.* rings, trophozoites and schizonts were present). After infection huRBC and clo-lip were supplied every 3 days as described for the pre-infection step. In some experiments, 250 mg/kg of inosine (Sigma) was injected intraperitoneally every day as the half-life of inosine is very short [20].

In experiments using NSG mice the protocol has been adapted in order to achieve varying levels of adequate huRBC chimerism and to avoid overloading the mice. As such, different amounts of blood were employed, and either 200, 400, 550 or 750 µl huRBC was injected 3 times per week (i.e. Monday, Wednesday and Friday) mixed with 250 µl human AB serum, as it has previously been described that human serum improves huRBC survival in immunocompromised mice [11]; 4 injections were done pre-infection, and clo-lip was injected as described above. Follow-up of the infection was performed by daily Giemsa stained thin blood films drawn from the tail vein.

### 5. Mouse cell isolation

In NOD/SCID mice inflammation was induced by IP injection of 1 ml 3% thioglycolate (Sigma) diluted in sterile PBS. 4–5 days after, the peritoneal cavity was washed with HBSS without Ca^2+^ and Mg^2+^. The collected cells were washed twice in RPMI supplemented with L-glutamine, Penicillin (100 U/ml), Streptomycin (100 µg/ml), and 10% Fetal Calf Serum (FCS) and seeded at 3×10^5^ per well in a 96-well culture plate. Single cell suspensions of splenocytes were prepared in cold RPMI 1640 medium supplemented with 10% FCS and filtered on a 100 µm cell strainer to remove debris. Erythrocytes were lysed with ACK lysis buffer, and the splenocytes were washed 2 times with RPMI supplemented with 10% FCS and seeded at 3×10^5^ per well of a 96 well culture plate.

### 6. Cytokines/chemokine/chemiluminesence assay

100 µl blood samples were collected from the retro-orbital plexus with a Pasteur pipette, and sera were stored at −80°C. Conditioned media obtained after 16 h stimulation of peritoneal cells and splenocytes with lipopolysaccharide (LPS, 1 µg/ml) (Sigma) were stored at −80°C. Cytokines and chemokines (IL-6, MCP-1, IFNγ, TNFα, IL-12p70 and IL-10) were quantified using the BD™ Cytometric Bead Array mouse inflammatory kit (BD biosciences) following the manufacturer's recommendations on a FACScalibur (BD biosciences).

Since production of reactive oxygen intermediates (ROI) closely mirrors the state of activation of macrophages and polymorphonuclear cells, luminol dependent photometric assay was used to measure ROI. Blood samples were collected from NOD/SCID mice, washed with HBSS with freshly added Ca^2+^ and Mg^2+^. Washed blood was diluted 1/10 in HBSS and 90 µl blood was added to each well in a 96 well plate (Nunc, Denmark) and incubated for 30 minutes at 37°C after adding 10 µl PMA (final concentration 1 µg/ml) to stimulate the cells. 50 µl of luminol (final concentration 200 µg/ml) solution was added immediately before measuring emissions.

### 7. Analysis of deep-seated organs for parasite differential count

NSG mice were used for the comparison of parasite differential counts in the peripheral blood, with that in deep-seated organs. Four mice were infected with UPA strain, and when a parasitemia of >10% was reached, a thin smear from peripheral blood was made before killing the mouse, and harvesting its organs. Kidney, liver, spleen, lung, and brain were removed from each mouse. Parasite content was assessed from blots made by repeatedly spotting sections from each organ. These slides were then stained with Giemsa. The last blots taken were considered to be the most representative of the parasite content in the organ's vascular bed, and were examined at 1000x magnification to perform differential counts of each stage (>200 parasites from each organ counted).

## Results

### Development of an IV model

**Figure 1:**
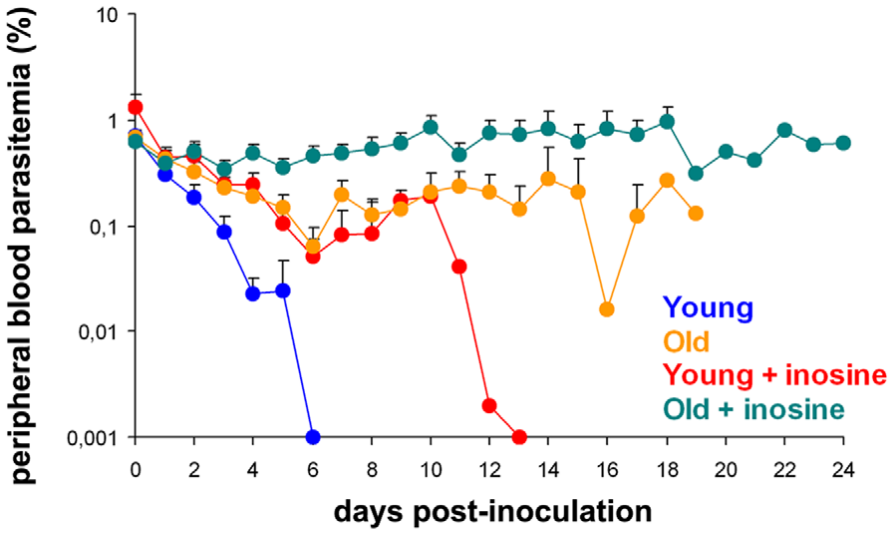
Effects of aging and inosine on parasitemia in NOD/SCID mice. Parasitemia is shown as a percentage of total erythrocytes found in mouse peripheral blood measured on giemsa-stained thin smears. Levels are shown for old mice (>15 weeks) receiving inosine (green) or without inosine (yellow) and young mice (<15 weeks) with inosine (red) or without (blue). *P. falciparum* 3D7 strain was inoculated on day 0. A 400 µl pellet of huRBC and clo-lip (100 µl) were administered (IV and IP respectively) 3 times a week to all mice. For the inosine groups, inosine (250 mg/kg) was administered IP daily. Results represent the mean ± SEM from 11 different experiments, positive result/success means parasitemia lasting >12 days a) green curve: n = 41, 15 positive, 36,6% success, b) yellow curve; n = 30, 5 positive, 16,6% success; c) red curve; n = 29, 0 positive, 0% success; d) blue curve; n = 10, 1 positive, 10% success.

In a previous study [6], we have shown that among NOD/SCID mice inoculated intraperitoneally with huRBC and *P. falciparum* (IP-IP model), the majority do not support a stable and sustained peripheral blood parasitemia. For this reason we attempted to develop a new model in which both huRBC and *P. falciparum* are directly injected into the mouse blood stream (IV-IV model). With this IV protocol 100% of NOD/SCID mice proved to be parasitized by day 1 post-inoculation *vs* only 56% using the IP protocol (data not shown). Despite this marked improvement, among the 59 mice treated with the standard immunomodulatory protocol of innate defences (clo-lip), the parasitemia duration was short (average 5.5 days) and parasitemia was stable for ≥12 days in only 5 mice (Figure 1).

We sought to better understand this large variation in duration of parasitemia. It was not explained by differences in chimerism. However, close analysis did show a significant difference in parasitemia duration between young mice and aged mice.

### Effect of age on inflammation

We define young mice as those less than 15 weeks old and old mice as greater than 15 weeks old. While 16.6% of the aged mice were able to support a parasitemia lasting more than 12 days, none of the young mice supported a parasitemia lasting this long, and average length of parasitemia for old *vs* young was 7.1 *vs* 3.9 days respectively. The gender of the mice made no difference.

**Figure 2:**
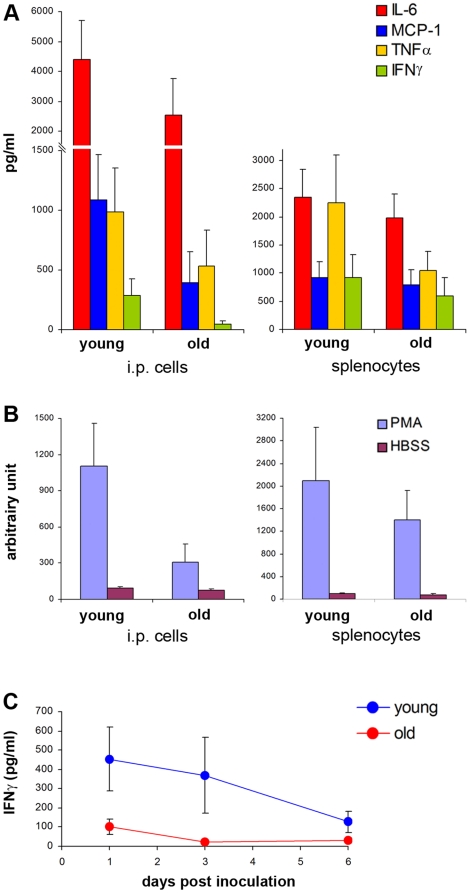
Effect of the aging process on the production of cytokines in NOD/SCID mice. Panel (A): levels of various inflammatory mediators (IL-6, MCP-1, TNFα, IFNγ) in supernatants of peritoneal cells (n = 10) and splenocytes (n = 16) from young *vs* aged mice after stimulation with LPS (1 µg/ml) ; Panel (B) : levels of reactive oxygen intermediates, measured by chemiluminescence, in the presence of PMA (10 µg/ml) produced by peritoneal cells (n = 4) and splenocytes (n = 5) taken from young *vs* aged mice; Panel (C): *in vivo* levels of IFNγ in aged mice (with long-lasting parasitemia) (n = 4) compared to young mice (with short-lived parasitemia) (n = 5) as measured in serum samples collected 1, 3, and 6 days post infection with *P. falciparum.* Cytokine levels were measured by using the CBA mouse inflammatory kit by FACS. Results represent the mean ± SEM from two different experiments.

To explain this phenomenon, we analysed the inflammatory responses of healthy non-infected “young” and “aged” NOD/SCID mice. The levels of inflammatory mediator production were investigated in peritoneal cells and splenocytes from these mice *in vitro* after stimulation with LPS for 16 hours. The levels of IL-6, MCP-1, TNFα and IFNγ were 1.7, 2.8, 1.8, and 6.4 times lower in the supernatant of peritoneal cells from aged mice as compared to that from young mice, respectively. Despite these important differences, probably due to group size, they were not statistically significant. The same investigation was performed using splenocytes, and the findings were more modest. The levels of IL-6, MCP-1, TNFα and IFNγ were 1.18, 1.16, 2.14 and 1.54 times lower in supernatants of splenocytes from aged mice as compared to young mice, respectively (Figure 2A). Interestingly, cells from young mice produce very strong inflammatory responses when incubated in serum from young mice, whereas these responses are decreased when they are incubated in serum from old mice. This would imply that these age-related differences are mediated by humoral factors.

Furthermore, a study of O2- free radical emission from PMA-stimulated peritoneal cells detected by chemiluminescence showed major differences for young *vs* aged mice cells, i.e.: 1104±354 *vs* 308±152 CU respectively, and in splenocytes 2100±941 *vs* 1398±528 CU respectively. These findings were not statistically significant either, however (Figure 2B).

The same was addressed in *P. falciparum* infected NOD/SCID mice using serum collected 1, 3 and 6 days post-inoculation, either from young mice with short-lived parasitemia or aged mice with long-lasting parasitemia. Among the cytokines/chemokines measured only IFNγ differed, particularly on days 1 and 3 (102±38 *vs* 454±168 at day 1, and 24±8 *vs* 369±196 at day 3, in aged *vs* young mice, respectively) (Figure 2C).

### Effect of immunosupressants on NOD/SCID mice

In order to improve sustainability of parasitemia in the mouse model further, we investigated immunosuppressive agents that might help to minimize the innate immune response. Inosine, a purine nucleoside, proved to be the most effective agent at improving peripheral blood parasitemia from a group of agents tested, including TGFβ1, dexamethasone, and IL-10.

**Figure 3:**
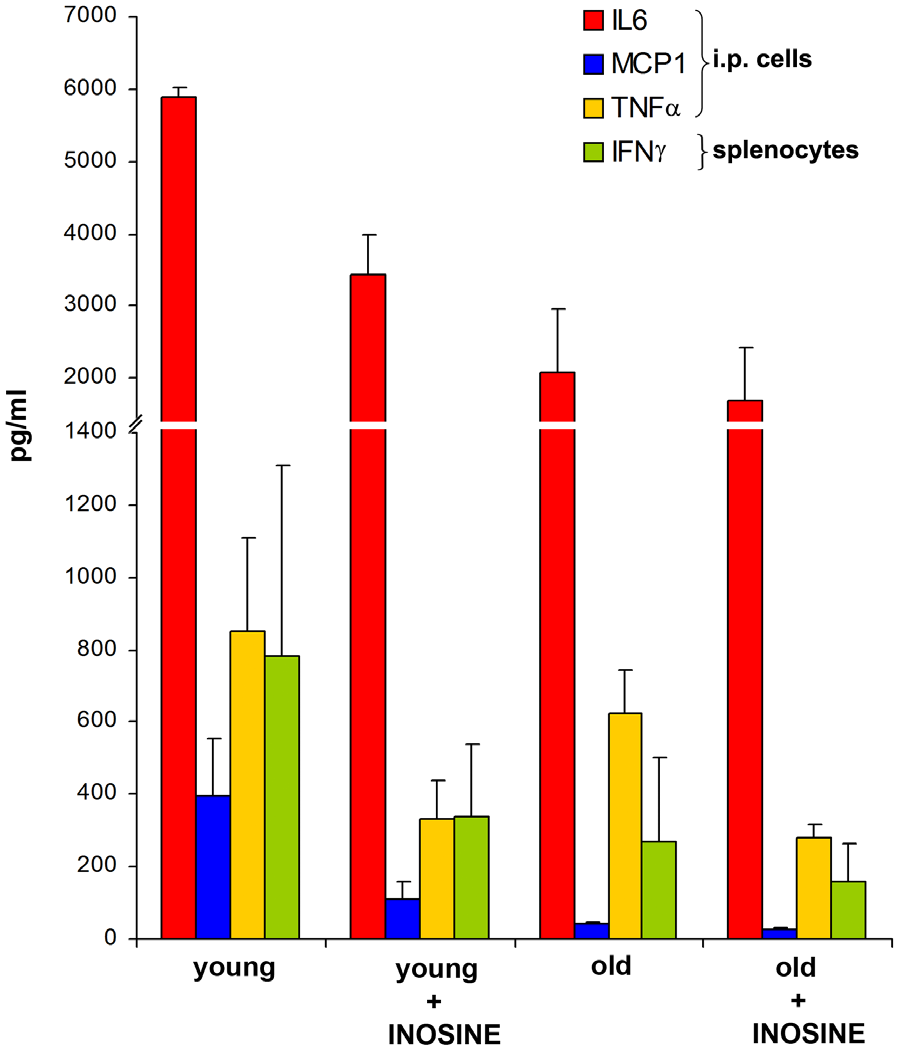
Effect of inosine and the aging process on production of inflammatory mediators in NOD/SCID mice. Determination of levels of cytokines in supernatants of peritoneal cells (IL-6, MCP1, TNFα) or splenocytes (IFNγ) collected from NOD/SCID mice and stimulated *in vitro* with LPS (1 µg/ml). Results from cells of aged mice (>15 weeks) and young mice (<15 weeks) incubated with or without 1 mM inosine are shown. Results represent the mean ± SEM from an experiment performed using 3 mice per group.

*In vitro* experiments found that at a concentration of 1 mM inosine decreased IL-6, MCP-1, and TNFα secretion from young NOD/SCID LPS stimulated peritoneal cells by a ratio of 1.72, 3.55, and 2.56 respectively as compared to similar inosine-untreated cells. This effect was less pronounced on cells from aged mice, with respective decreases of 1.23, 1.49, and 2.24, although age related differences were not significant (Figure 3). The other cytokines/chemokines tested (IL-12p70, IFNγ and IL-10) remained unchanged. In the case of NOD/SCID mouse splenocytes, only IFNγ release was decreased by treatment with inosine *in vitro.* The reduction of IFNγ production in young and aged mice was 2.3 and 1.7 times respectively.

*In vivo* inoculation of inosine confirmed the above data in both young and aged mice. In young NOD/SCID mice daily intra-peritoneal injection of inosine at a dose of 250 mg/kg led to 10% of mice sustaining parasitemia for >12 days, as compared to none in the inosine-free group. Using the combination of aged mice and inosine administration resulted in 37% of mice sustaining parasitemia for >12 days, and an average length of parasitemia of 9.8 days.

### Development of an improved IV model using NSG mice

Experiments were performed in NSG mice using various amounts of huRBC (200, 400, 550 or 750 µl of RBC pellets) injected IV 3 times a week, and also using the IV route to introduce the parasite infection. Clo-lip was delivered IP on the same day as huRBC administration. This is called the NSG-IV model below.

**Figure 4:**
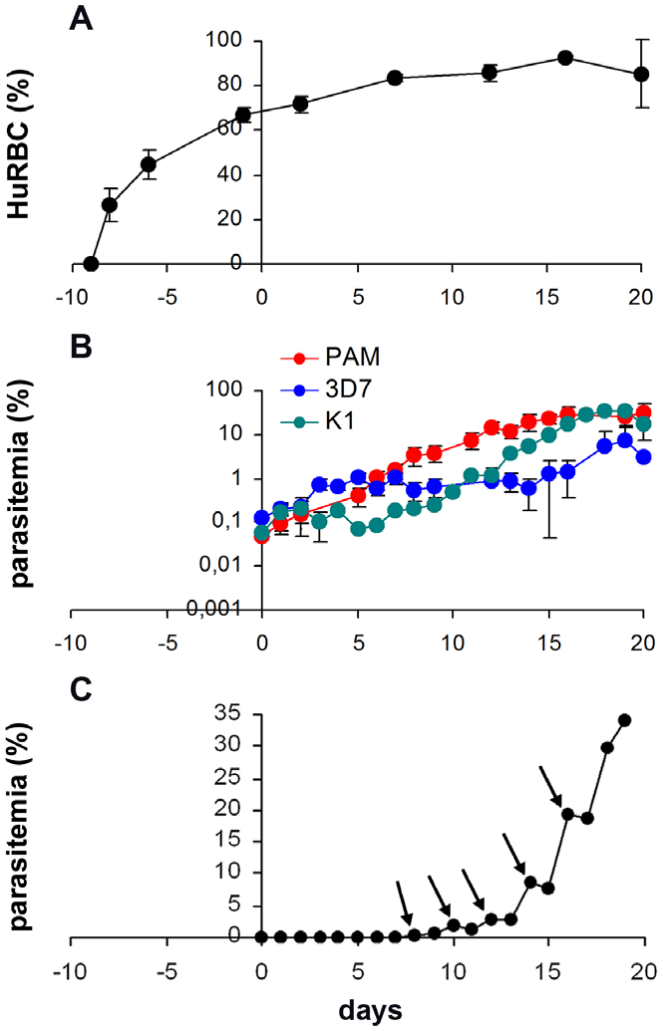
Results obtained using the NSG-IV *P. falciparum* model. Panel (A): proportion of huRBC (% of total erythrocytes), or “chimerism” seen in peripheral mouse blood, as determined by FACS analysis using an anti-human glycophorin A antibody (average of 18 mice). HuRBC grafting started 10 days before infection with *P. falciparum*; Panel (B): parasitemia patterns obtained after inoculation with either UPA (red; n = 10), 3D7 (blue; n = 4), or K1 (green; n = 4) *P. falciparum* strains. Results represent the means ± SEM from 2 different experiments. Panel (C): detailed pattern of *P. falciparum* growth over time in the NSG-IV model. Arrows indicate points at which >95% of parasites were at ring stage. Parasitemia is expressed as a percentage of total erythrocytes found in mouse peripheral blood. Mice received 750 µl of huRBC delivered IV 3 times a week, along with IP administration of liposomal clodronate (100 µl).

**Figure 5:**
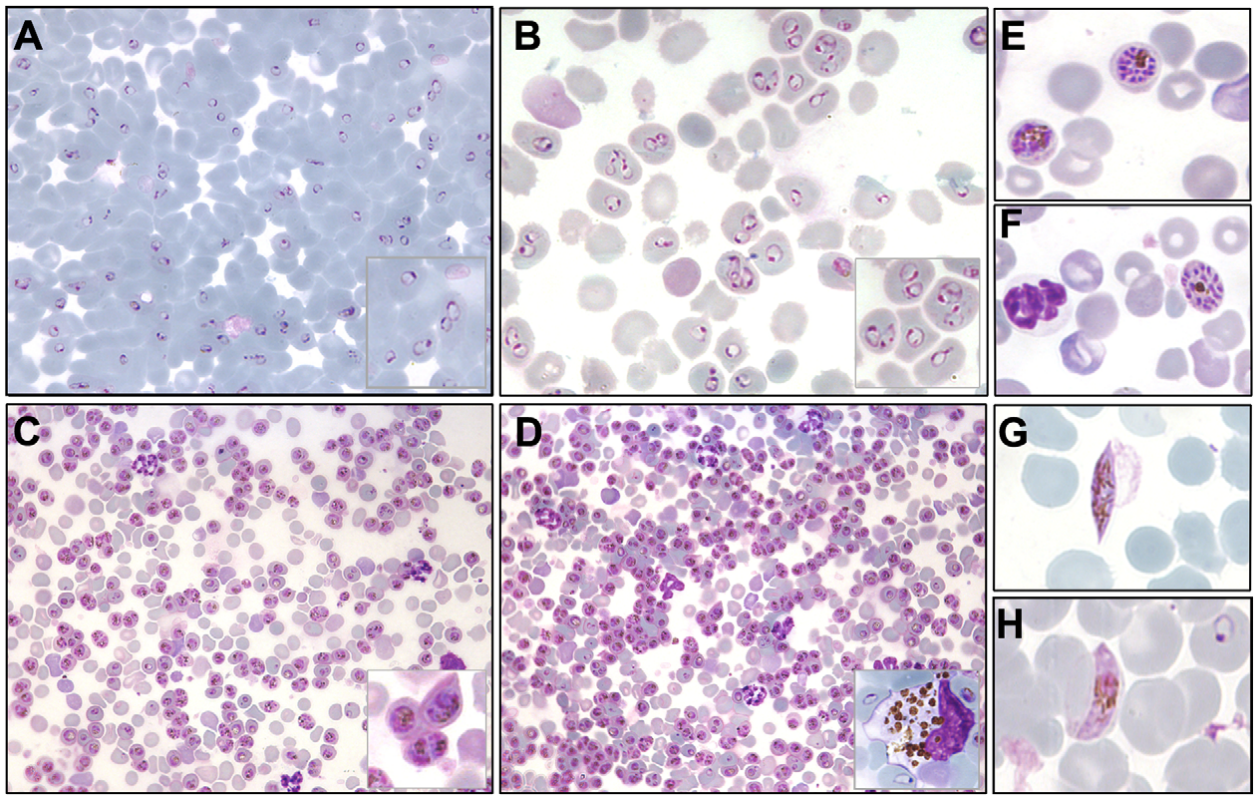
Examples of parasitemia obtained in the NSG-IV model. Panels (A) and (B) illustrate the typical predominance of ring stage parasites seen at 48 h intervals (see Fig. 4 C and SI-II), with frequent polyparasitism. Such parasite densities are common using UPA parasites in mice having >75% chimerism. Panel (C) fully mature trophozoites seen 24 hours later, as compared to A and B. Single nucleus trophozoites predominate, though some can be mistaken for schizonts due to polyparasitism. However rings and young schizonts are also present in small proportions. Panel (D) illustrates one of the highest parasitemias obtained. Insert: active phagocytosis by a circulating monocyte. Panels (E) and (F) illustrate fully mature schizonts which are only occasionally seen in peripheral blood. Panel (G) stage IV gametocyte, Panel (H) rare occurrence of mature gametocyte (stage V) in peripheral blood.

Injecting 750 µl of huRBC resulted in a high proportion of huRBC in mouse peripheral blood (chimerism), ranging from 83% to 92% of total erythrocytes (Figure 4A). This high level of chimerism was stable over a number of months and supported rapid, optimal growth of *P. falciparum* (Figure 4B) with abundant and very healthy parasites (Figure 5). Indeed peripheral blood parasitemia levels reached 30–35% within 3 weeks (Figure 4B) and went on to reach a maximum of 56.2% (which corresponds to a parasitemia of 67% in the huRBC subset of mouse peripheral blood). The total haematocrit in mouse peripheral blood was on average 70% using this protocol (data not shown).

**Figure 6:**
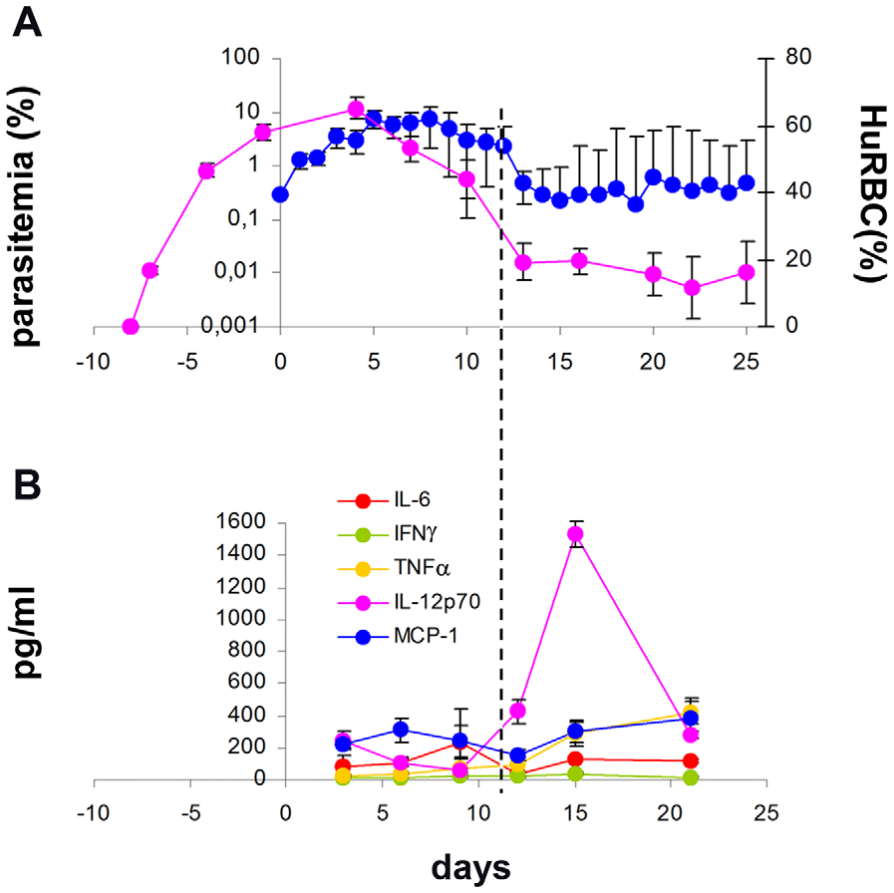
Residual inflammation present in the IV-NSG model. Panel A: using the sub-optimal dose of 400 µL huRBC grafting results in a reduction of chimerism (red line) and parasitemia (blue line); Panel B: inflammatory cytokine responses were measured in parallel using the CBA kit. Note the initial rise of IL6, followed by a major increase of IL-12p70, and a modest increase of TNFα and MCP-1 that are temporally associated with anemia, and might play a role in its etiology. Results represent the means ± SEM from 2 experiments (n = 7).

Use of a lower dose of huRBC (400 µl) resulted in an initial chimerism of about 60% (Figure 6A), and parasitemia of up to 7.2% (ca. 12% of huRBC parasitized). Use of this lower huRBC volume however, was followed by a decrease in chimerism most likely due to inflammation induced by the parasite, producing a parallel reduction in parasitemia, which fluctuated at an intermediate level, i.e. without complete parasite clearance (Figure 6B).

An analysis of inflammatory markers was performed in mice from the latter protocol to investigate the observed decrease seen in chimerism. The results revealed substantial differences from NOD/SCID mice. The decreased huRBC chimerism and parasitemia seen in NSG mice correlated with the release of TNFα and IL-12p70. In contrast IL-6 and IFNγ remained almost unchanged (Figure 6B), whereas in NOD/SCID mice IL-6 and IFNγ levels increased at the time of parasite decrease [6]. Hence the inflammation produced by NSG mice is reduced in comparison to that seen in NOD/SCID, and this is likely to be one of the reasons for improved *P. falciparum* survival.

We observed from the variety of protocols experimented with, that provided the chimerism is maintained above 10–20%, parasitemia can be supported for a number of months (i.e. the mouse life-span). So, to avoid volume overload in the mice, intermediary protocols were designed requiring lower volumes of IV administered huRBC: i) satisfactory results were achieved in all mice where 550 µl of huRBC was injected three times a week (n = 47), with a mean ± SD chimerism of 74%±7% over the first month and half, decreasing to 56% over the second and third month, and long-lasting parasitemia reaching up to 20–40% (25.6% to 51.3% of huRBC parasitized); ii) when 550 µL of huRBC was injected only twice a week, a chimerism of 40–60% was achieved and this supported a moderate 5%–12% parasitemia during the first month.

In this manner, adjusting the quantity of huRBC injected leads to various levels of prolonged, stable parasitemia. The level can be tailored in this manner to the needs of each experiment.

In this model there was no need for preliminary adaptation of the parasites, contrary to what has been reported for another model based upon the NSG mouse [11]. All strains tested were supported (UPA, 3D7, K1), and parasite growth was readily apparent from the first days post-inoculation. Naturally, there were some variations in the maximum parasitemia reached with each strain, particularly for 3D7, which produced the lowest. Mice were also infected with a fresh clinical isolate of *P. falciparum*. With this isolate parasitemia was uniformly composed of young ring forms for the first 4 days, then trophozoites appeared as the parasitemia rose from 0.1 to 1%. Each of the 2 animals infected showed satisfactory growth as did mice sub-inoculated with parasites from the initial animals (not shown).

Mice used in the NSG-IV model, when the 550 µl dose is given, demonstrated a life expectancy equivalent to, or slightly better than that seen in NOD/SCID mice used for previously described models (i.e. 2–3 months).

The NSG-IV model was reliably reproducible. All mice grafted with huRBC and infected with *P. falciparum* to date developed a peripheral blood parasitemia (n = 68). The degree of chimerism and parasite growth was consistent and homogenous from one animal to the other (see Figure 4B). This is a critical difference from the SCID, BXN or NOD/SCID models [6], [7], [8], [10].

To confirm the advantage of the IV route, the same NSG mice were used in a protocol using intra-peritoneal (IP) grafting of huRBC, and IP infection with *P. falciparum.* Despite the advantage of a decreased inflammatory response in this strain of mice, this “IP-IP” protocol provided erratic outcomes, with large individual variations from one animal to the other in terms of presence of parasites in peripheral blood (data not shown). Ultimately, the IP-IP protocol in NSG proved to be as inadequate, and as troublesome as the same route in NOD/SCID.

The value of inosine was re-addressed in NSG. In experiments performed using the 400 µl huRBC dose, inosine was administered IP on a daily basis in addition to the standard use of clo-lip. While the benefits of inosine on parasite survival were initially not as obvious as in NOD/SCID mice, statistically significant improvement of parasitemia in NSG mice receiving inosine (n = 3), as compared to NSG without inosine (n = 4), became apparent (Figure S1) after about 2 weeks of treatment, i.e. when strong inflammatory responses are induced by high parasitemia (non parametric Wilcoxon test on 55 paired values: Z = 3.65, p value = 0.0003).

### Additional characteristics of *P. falciparum* in the NSG-IV model

Remarkably, parasite growth occurred in a synchronous fashion (Figure 4C and Figure S2), with the vast majority of parasites in peripheral blood being at the same stage at a given time (Figure 5). As shown in Figure 5 A, B, C, D, over 95% of parasites were simultaneously either at young ring stage or at trophozoite stage. This resulted in a stepwise increase in parasitemia. As demonstrated in Figure 4C, over the first 24 hours the parasite matures without an increase in parasitemia. This is then followed the next day by the reinvasion of new huRBC, leading to a steep increase in the proportion of infected erythrocytes (Figure 4C and Figure S2). As asynchronous parasite cultures were used to infect animals, this suggests a synchronizing effect of the mouse innate defences. This is the first-ever animal model for *P. falciparum* that exhibits a synchronised asexual blood stage cycle as occurs in human beings. However this characteristic was not constant, in particular when there was long-standing high parasitemia; this synchronism eventually disappeared in some of the mice as illustrated in Figure S3.

Fully mature schizonts, i.e. segmenters, were seen very rarely in peripheral blood and this occurred mostly at high levels of parasitemia. However, the high rate of re-invasion implied that they were developing and releasing merozoites in a synchronised manner elsewhere.

To investigate this further we analyzed the parasite stages in peripheral blood, as compared to those in deep-seated capillary beds from various organs. In peripheral blood segmenters were only seen rarely when parasitemia was low, particularly during the initial increase following infection of mice, i.e. <1% of all parasites detected. When the parasitemia increased, their proportion slightly increased, i.e. reaching 3–5% of those huRBC parasitized. Among all parasites seen, the majority were rings or very mature pigmented trophozoites (parasites at a single nucleus stage). The very mature trophozoites seen did not differ in morphology from similar stages that can be appreciated in peripheral blood from a malaria patient. The simultaneous presence of 3–4 mature trophozoites in the same RBC (Figure 5C and D) sometimes can be mistaken for a schizont. Schizonts were present however, but were only seen rarely.

The parasite load in blood from various organs investigated was similar to that seen in peripheral blood at the time of sampling. However capillary blood from the brain, kidney, spleen, and liver revealed a 4–5 fold higher proportion of segmenters (detailed counts of brain and kidney showed that 14–16% of parasites were fully mature schizonts, at a time when the peripheral blood contained only 3% of them). Tissue sections examined by IFAT using an anti-HSP antibody also revealed numerous pigmented mature forms in the organs; however it was not possible to perform a differential count as rings were hardly identified at all by this means (not shown). These findings are suggestive of an intermediate level of sequestration in the NSG-IV model, with high numbers of very mature trophozoites and occasional presence of schizonts in peripheral blood, but with markedly higher prevalence of mature schizonts in organ vascular-beds.

Gametocytes were also able to develop in the NSG-IV model. These sexual forms (Figure 5G, H) were occasionally seen up to stage III or IV, and developed rarely to stage V. The observation of stage V gametocytes implies that infected huRBC are able to survive in the mouse circulation for, at least, 9 days without suffering any significant immune-mediated insults when the NSG-IV protocol is used.

## Discussion

This study is part of our longstanding effort aimed at creating a satisfactory experimental mouse model for *P. falciparum* malaria. By employing the IV route for both parasite and huRBC delivery, liposomal-clodronate for macrophage suppression, and the IL-2Rγ mutation on the NOD/SCID genetic background, we have achieved a model that has 100% reproducibility, is receptive to numerous strains without requiring pre-adaptation of the parasite, and supports fast-rising, high-reaching *P. falciparum* parasitemia that remains stable for weeks or months.

Results also show that the pro-inflammatory effect of *P. falciparum* is better controlled, however still suboptimally. In addition to clodronate encapsulated liposomes (clo-lip) [6], we now demonstrate, in NOD/SCID mice, the immunomodulatory effects of aging, of inosine and of the IL-2 receptor γ mutation. The latter brings the greatest breakthrough with a marked reduction in inflammatory cytokines/chemokines and, most likely for this reason, improved *P. falciparum* survival. Results are in keeping with those reported recently using an hybrid IP-IV model and an immunodeficient mouse-adapted *P. falciparum* strain [14]. The combination of an IV-IV protocol and prudent use of immunosuppressive factors has produced a model that has a number of attractive features over those previously described.

The main advantage is an extremely high success rate, with 100% of mice that were infected showing a healthy *P. falciparum* parasitemia. This represents a dramatic improvement compared to the low proportion of mice with stable parasitemia that we obtained in previous models [6], [8]. By using the IV route and clo-lip we reduced huRBC requirements and above all excluded the unpredictable, poorly understood transperitoneal passage of huRBCs as employed in existing models [6], [11], [21].

Another advantage is the high reproducibility. The parasitemia curves were very consistent from one animal to the other with low standard deviations, a feature that will be important when using the model for vaccine or drug studies. This level of consistency is, in fact, more so than what is seen in human infections where untreated cases show day-to-day variations in parasitemia by one degree of magnitude or more [22]. The parasitemia is also capable of reaching high levels of density, particularly when calculated as a percentage of the huRBC subset rather than total erythrocytes. These favourable characteristics of the model are complemented by a rapid rate of parasite growth, which rises faster than in other models [6], [9], [21], with very healthy looking parasites and frequent polyparasitism of huRBC.

Several results concur to suggest that the model is closer to events recorded in humans than other existing *P. falciparum* models, namely receptivity to non-adapted parasites, synchronisation, partial sequestration of mature forms and gametocyte production.

All *P. falciparum* strains tested so far have been supported, indicating that, in contrast to *Aotus, Saimiri* and previous mouse models, there is no requirement for preliminary adaptation of the parasite to this new host [11], [14], [23], [24], [25]. This was true for the 3 strains employed and for one clinical isolate of *P. falciparum* tested. Results obtained with the latter bode well for the potential of the model to support *P. vivax* growth.

This is the only Malaria model that has ever demonstrated the phenomenon of synchronisation, which is not seen either in *Aotus*, *Saimiri,* or other immunodeficient mice [11], [14], [23], [24], [25]. Examination of slides is very impressive with over 95% of parasites seen being at the same stage. This synchronisation results in a very characteristic step-wise increase in parasite density at 48 hour intervals - reminiscent of tertian Malaria - at least during the initial rising phase. The various stages seen in the peripheral blood do not differ morphologically from those that are observed in human peripheral blood smears, although very high densities of mature trophozoites can be seen in NSG which, obviously, is seldom seen in humans.

Partial sequestration takes place with an overwhelming dominance of rings or mature trophozoites (i.e. parasites at one nucleus stage) in peripheral blood, and conversely rare schizonts. However the presence of schizonts seems to increase at high parasitemia. Examination of blood from deep-seated capillary beds from various organs shows that the most mature segmenters are far more frequent, ca. 4–5 fold, in blood from the brain and the kidney, than in peripheral blood. This partial sequestration could result from sub-optimal affinity of the *P. falciparum* infected huRBC for the heterologous murine endothelium. Conversely mature forms are also occasionally seen in human peripheral blood particularly in severe cases at high parasite loads [26]. Thus, it is also possible that at high parasite loads, phagocytosis of numerous parasites by neutrophils results in neutrophil necrosis, causing release of neutrophil proteases, which together with parasite proteases, may in turn contribute to desequestrate mature forms from the endothelium [27]. Whatever the case, the picture that emerges is one of intermediate, rather than absolute sequestration. Further work will be required to determine if the model can help understand the sequestration phenomenon which is characteristic of *P. falciparum,* and to see if it has relevance to the human situation.

The model also proved to support the development of the *P. falciparum* sexual blood cycle. Stage III and IV gametocytes could be seen in the peripheral blood; however stage V gametocytes were rarely seen, most likely as a result of the high turnover rate of huRBC caused by the residual inflammatory response, and the long duration needed for the sexual blood cycle.

From a practical point of view the described NSG-IV model is very flexible, and levels of parasitemia can be adjusted, by modulating the amount of huRBC injected, to anywhere from 1–2% to 30–40% in order to meet the needs of the experiment, and of the investigator. The model remains demanding however, with 2–3 IV inoculations required per week.

Results also show, that innate defences are still very active and hence that the model remains suboptimal. Detailed analysis better defined the role of each of the cytokines/chemokines produced in this process, and helps to identify which ones need to be controlled to improve success. Our findings contribute in this way to the understanding of the delicate balance between inflammation control and *P. falciparum* survival.

The relationship between aging and the mouse's ability to both tolerate the graft and produce less inflammatory mediators is an interesting finding that emerged from the analysis. Aging is well known to be associated with increased susceptibility to infections [28], [29]. There is currently a renewed interest in the effect of age on the immune system, both adaptive and innate. Several recent studies have described modifications of immune function in aged subjects, leading either to increased, or more frequently decreased inflammatory processes with a reduction in LPS-induced IL-6, and IFNγ levels, similar to what we observed [30], [31], [32], [33]. However we are not aware of any previous data pertaining specifically to NOD/SCID mice. The present findings of improved graft survival associated with a decreased release of inflammatory cytokines by macrophages in “aged” mice complements previous findings in other mouse strains [34], and indicates that this process reduces defences against *Plasmodium*. The effect of aging was not evident in the NSG mice however. This is likely to be due to a blurring of the effect by interposition with the IL-2Rγ mutation, along with a sample size too small to show a marginal benefit of aging in these mice.

Among a large number of immunomodulating substances investigated, inosine emerged as one of the few drugs beneficial in this scenario. It is well known that adenosine possesses anti-inflammatory properties [35], and that its derivative analogues such as inosine, can provide protection to mice from a variety of inflammatory diseases including endotoxic and septic shock [35], [36], acute lung injury [20], [37], pleural inflammation [38], and colitis [39] by inhibiting the release of free radicals and pro-inflammatory cytokines [40]. Inosine, a naturally occurring purine, and breakdown product of adenosine by adenosine deaminase [41], has been shown to have both pro and anti-inflammatory properties, probably mediated by its ability to bind to more than one type of adenosine receptor. The results of the present study showing that inosine reduces the release of IL-6, MCP-1 and TNFα by macrophages from NOD/SCID mice, is consistent with previous reports using cells from immunocompetent mice [42]. These effects are supported by the significant improvement that was seen in parasitemia for mice that received inosine, compared to those that did not. Inosine may also act through its ability to suppress the function of neutrophils. It has been reported to inhibit neutrophil recruitment *in vivo*, and inhibit neutrophil superoxide production *in vitro* [40]. While the effect of increasing age on an NSG background was not evident, the benefit of administering inosine was significant at high parasitemia, i.e. after 15 days of treatment.

The relationship observed in this study, as in our previous study [6], between the peak of inflammation and a reduction in huRBC count should be stressed. It suggests that the anemia seen correlates with increased inflammation, and that it could potentially be the inflammation itself that causes the anemia, independent of the parasite, through destruction of non-infected huRBC. This might be mediated by free oxygen radicals damaging the huRBC membrane [43]. In this respect the model may provide novel research hypotheses that could lead to better characterize the poorly understood pathophysiological process of severe anemia in humans, which is one of the two major causes of mortality [44], although admittedly the human situation differs substantially from that of *P.falciparum* in the NSG mice.

Our results confirm that the NSG mouse strain has a significantly attenuated inflammatory response, leading to an improvement in parasitemia. These mice have proven to be excellent hosts for the grafting of various human cells, and notably they are better recipients than NOD/SCID for CD34+ hematopoietic stem cells and human leukocytes [15], [18], [45]. This improved receptivity results from the reduced function of macrophage and dendritic cells, in addition to the lack of T, B, and NK cells [18], [45]. The improved survival of *P. falciparum* in NSG was associated with an absence of significant production of the two major inflammatory cytokines IL-6 and IFNγ, whereas both were elevated in the NOD/SCID model [6]. This is not to say that the inflammatory response was quashed completely in the NSG strain. The need for further efforts to control the deleterious responses of the murine immune system in this model remains, likely by including additional genetic modifications, for example, by knocking out CCR2 on an NSG genetic background to reduce the egress, the recruitment and the number of inflammatory monocytes [46], [47], [48].

The improved survival of *P. falciparum* in NSG mice is in agreement with results recently reported by Jiménez-Díaz et al., where massive loads of huRBC were administered intra-peritoneally [14]. At the expense of injections on a daily basis of as much as 2 ml of blood (1 ml huRBC pellet) IP in 25 gram mice, and using a mouse adapted strain of *P. falciparum*, improved results as compared to NOD/SCID/β2m mice [11] were obtained. The fact that the mice were able to survive such severe treatment implies that there must have been massive intraperitoneal destruction, and intravascular hemolysis of grafted erythrocytes. Inflammatory mediator levels were not reported for this IP model however, and it is unclear how the huge huRBC loads operate. It has been reported in patients receiving blood transfusions, that large amounts of RBC can exert an immuno-suppressor effect upon macrophages [49]. It is also possible that macrophages overloaded by phagocytosis of an inordinate number of huRBC become unable to cope with additional parasitized cells. Similarly, by saturating the mouse with huRBC, a subset of them sufficient to permit *P. falciparum* development is more likely to remain intact, despite the massive amount of erythrocyte destruction. Whichever way, we repeated the protocol described by Jiménez-Díaz et al [14] and confirm their results (not shown).

The use of liposomes containing clodronate (clo-lip), as described in the macrophage “suicide” approach developed by Nico van Rooijen [50], [51] brings several advantages with respect to both parasite survival and the quantity of huRBC that need to be administered. The reduction in macrophage numbers induced by clo-lip, and its consequence on parasite survival has been described in previous work on NIH-III and NOD/SCID mice [6], [7]. In our NSG-IV murine model, clo-lip complemented the IL-2Rγ mutation very nicely in controlling inflammation, and thereby reducing erythrophagocytosis. For example, using this drug allowed us to reduce the total number of huRBC injected into mice by nearly 5 times as compared to the IP-IV model [14], i.e. a total of 1.6 ml *vs* 7 ml per week, per mouse (or nearly 10 times, i.e. only 0.8 ml *vs* 7 ml when a moderate parasitemia is desired), avoiding overload of the peritoneal cavity with [13] unreasonable volumes of fluid, proteins, membranes and the local pathological consequences.

In total, there are now three possible protocols that can be used in NSG mice, each with different outcomes. They differ in the routes of delivery used for huRBC and parasites - in the “IP-IP model”, both components are delivered intraperitoneally; in the model employed by Jiménez-Díaz et al. the former are delivered intraperitoneally while the infection is delivered intravenously leading to the “IP-IV model”; and in this study we demonstrate the “IV-IV model” where both components are delivered intravenously. The IP-IP model presents the same limitations as were described when using this protocol in NOD/SCID mice, with poor reproducibility of peripheral parasitemia. The IP-IV model remains effective at the expense of administering large amounts of huRBC to the mouse, daily interventions, and pre-adaptation of the parasite. Therefore, the IV-IV model seems to be more physiological, requiring lower amounts of huRBC and only 2–3 interventions per week. The requirement for 3 injections into the retro-orbital plexus per week is still burdensome, and should be replaced in the near future by a model harbouring a hematopoietic stem cell line capable of producing human erythroblasts/cytes [52], with the advantage of producing reticulocytes to also support *P. vivax* [53].

The main advantages of the NSG-IV model are that it offers extremely high reproducibility of both huRBC grafting and parasite survival, a rapidly rising parasitemia that reaches high levels, for long durations, and it does not need preliminary adaptation of parasite strains to the mouse. In addition, by modulating the quantity of huRBC administered, the final parasitemia can be adjusted to the needs of each experiment over a range of 1–40% for several weeks, or months, in a stable, controlled, and flexible fashion. Despite the improved behaviour and morphology of the parasites, there is still a residual inflammatory response that needs to be addressed in order to optimise this model further.

## Supporting Information

Figure S1 **Effect of inosine on the IV-NSG model.** Using the suboptimal dose of 400 µl of huRBC, which leads to a decrease in chimerism possibly due to inflammation as described in Figure 6, we show that the addition of inosine at a dose of 250 mg/kg per day IP (blue line) significantly improves the survival of *P. falciparum* in NSG mice compared to untreated controls (red line). Panel A: differences in chimerism; Panel B: differences in parasitemia on logarithmic scale; Panel C: differences in parasitemia on arithmetic scale. Statistical analysis using the non parametric Wicoxon ranking test of data from 3 mice (inosine) and 4 mice (controls) infected by UPA strain showing: from day 0–15 an absence of detectable effect (n = 15: Z = −1.43; P = 0,152), from day 15–29, a significant difference (n = 10: Z = −2,57; P = 0,0101), from day 27–67, a significant difference (n = 30: Z = 2.78; P = 0.0053), and for all days combined a very significant difference (n = 55; Z = −3.65; P = 0.0003).
(TIF)

Figure S2 **Further examples of synchronisation** **occurring during the initial phase of growth of** ***P.falciparum*** **in the IV-NSG mouse model, as shown by the curves of parasitemia recorded in 3 mice, which complement data presented in** **Figure 4** **.** Arrows indicate points at which >95% of parasites were at ring stage.
(TIF)

Figure S3 **Examples of asynchronous parasitemia obtained in the NSG-IV model.** Following several days at high parasitemia, the parasite cycle becomes asynchronous. In both panels, rings and trophozoites are concomitantly present, whereas schizonts remain rare.
(TIF)
