# Supplementary figures and images for: Further Improvements of the P. falciparum Humanized Mouse Model
Source: PLoS One. 2011 Mar 31;6(3):e18045. doi: 10.1371/journal.pone.0018045 (PMC3069031; doi:10.1371/journal.pone.0018045)

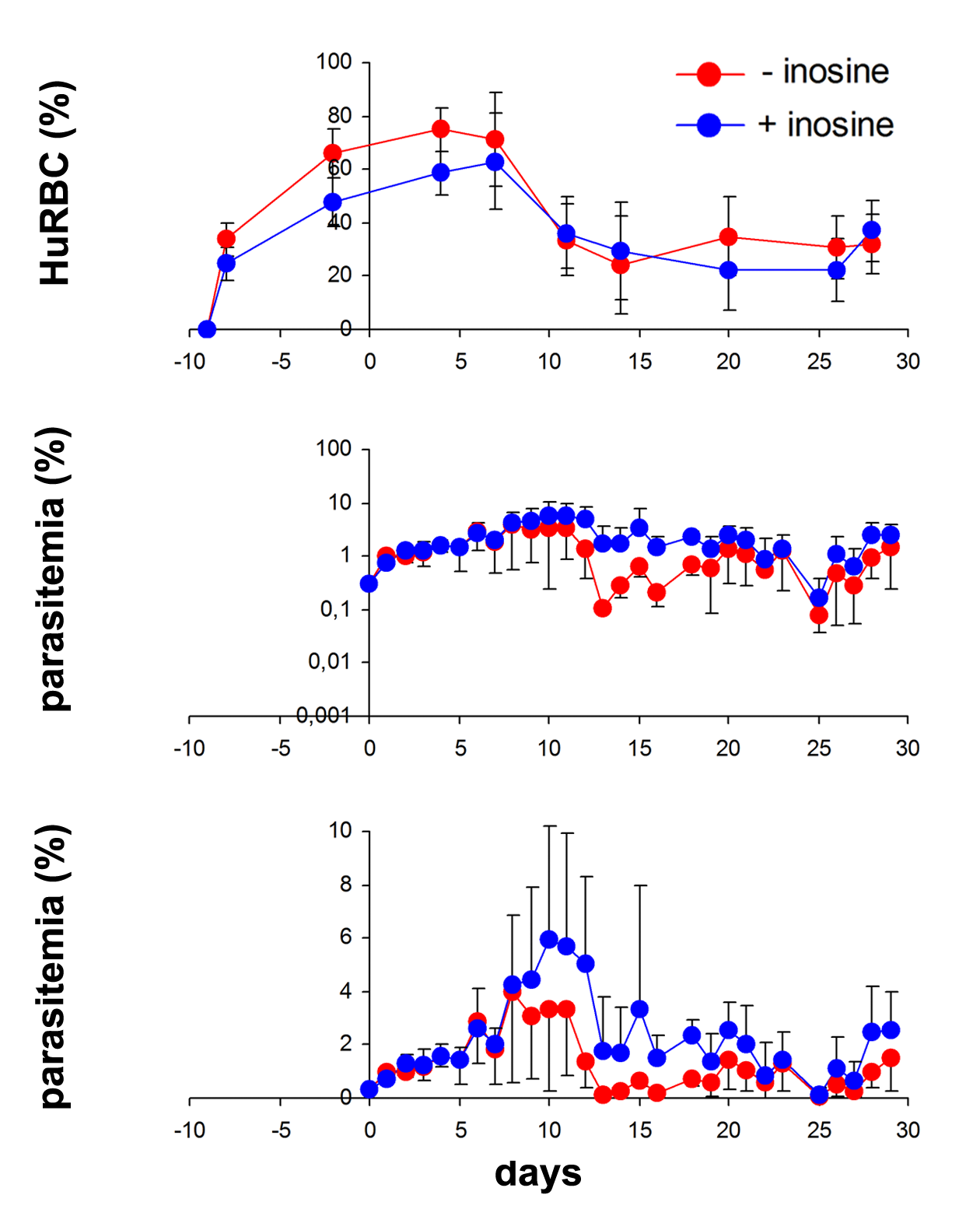

Supplement: Figure S1 — Effect of inosine on the IV-NSG model. Using the suboptimal dose of 400 µl of huRBC, which leads to a decrease in chimerism possibly due to inflammation as described in Figure 6, we show that the addition of inosine at a dose of 250 mg/kg per day IP (blue line) significantly improves the survival of P. falciparum in NSG mice compared to untreated controls (red line). Panel A: differences in chimerism; Panel B: differences in parasitemia on logarithmic scale; Panel C: differences in parasitemia on arithmetic scale. Statistical analysis using the non parametric Wicoxon ranking test of data from 3 mice (inosine) and 4 mice (controls) infected by UPA strain showing: from day 0–15 an absence of detectable effect (n = 15: Z = −1.43; P = 0,152), from day 15–29, a significant difference (n = 10: Z = −2,57; P = 0,0101), from day 27–67, a significant difference (n = 30: Z = 2.78; P = 0.0053), and for all days combined a very significant difference (n = 55; Z = −3.65; P = 0.0003). (TIF) [file pone.0018045.s001.tif]

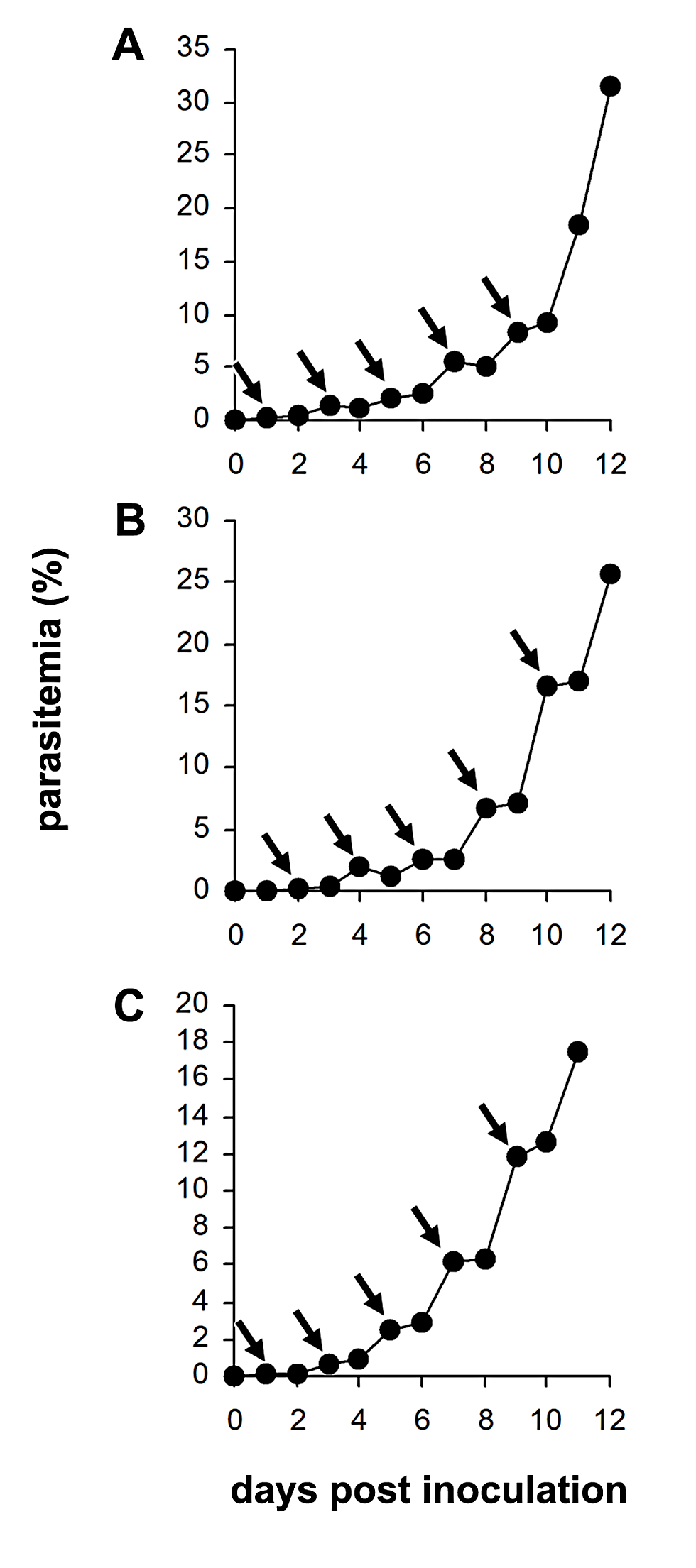

Supplement: Figure S2 — Further examples of synchronisation occurring during the initial phase of growth of P.falciparum in the IV-NSG mouse model, as shown by the curves of parasitemia recorded in 3 mice, which complement data presented in Figure 4 . Arrows indicate points at which >95% of parasites were at ring stage. (TIF) [file pone.0018045.s002.tif]

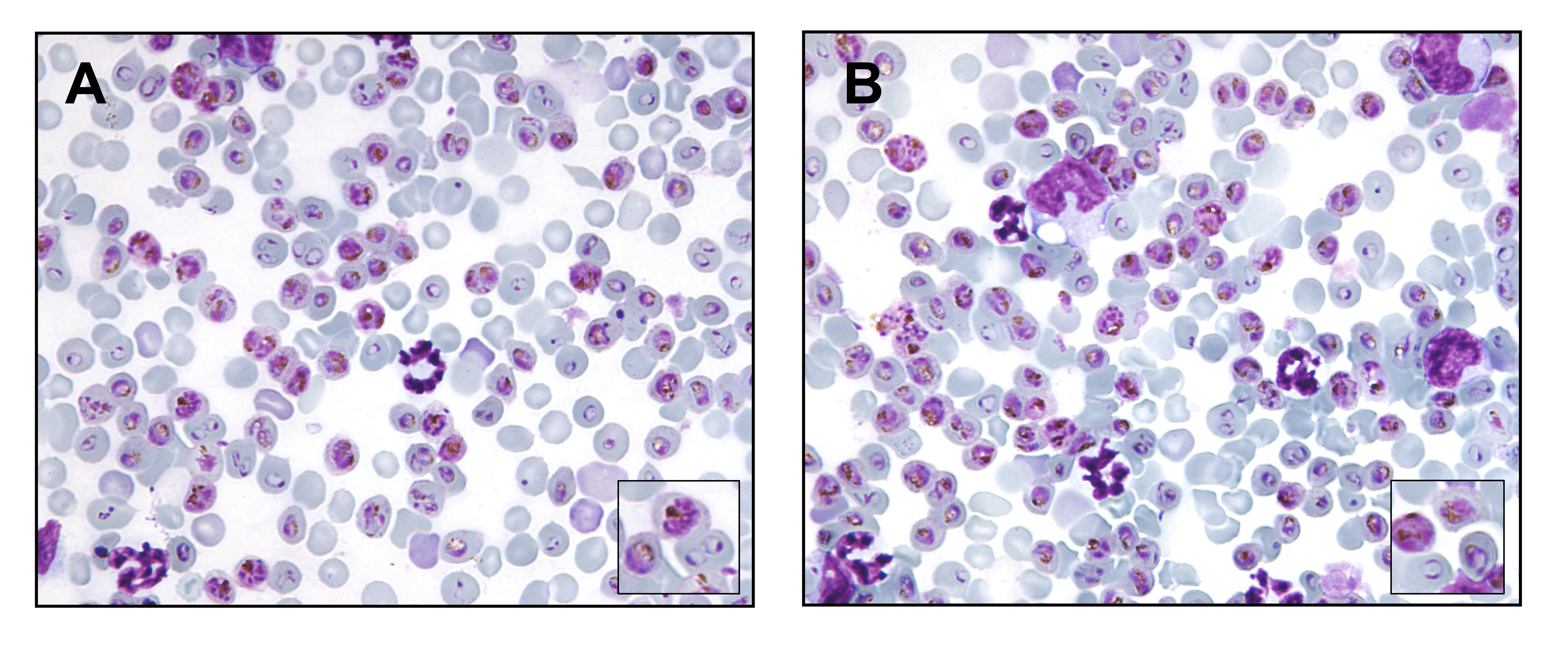

Supplement: Figure S3 — Examples of asynchronous parasitemia obtained in the NSG-IV model. Following several days at high parasitemia, the parasite cycle becomes asynchronous. In both panels, rings and trophozoites are concomitantly present, whereas schizonts remain rare. (TIF) [file pone.0018045.s003.tif]
